# Evaluation of point-of-care ultrasound training among healthcare providers: a pilot study

**DOI:** 10.1186/s13089-023-00350-5

**Published:** 2024-02-21

**Authors:** Dima Tareq Al-Absi, Mecit Can Emre Simsekler, Mohammed Atif Omar, Hatem Soliman-Aboumarie, Noha Abou Khater, Tahir Mehmood, Siddiq Anwar, Deanne Tomie Kashiwagi

**Affiliations:** 1 Department of Management Science and Engineering, Khalifa University of Science and Technology, Abu Dhabi, United Arab Emirates; 2 Department of Anaesthesia and Intensive Care, Harefield Hospital, Royal Brompton and Harefield Hospitals, Guy’s and St Thomas NHS Foundation Trust, London, UK; 3 School of Cardiovascular, Metabolic Sciences and Medicine, King’s College London, London, UK; 4 Department of Medicine, Sheikh Shakhbout Medical City, P.O.Box 11001, Abu Dhabi, United Arab Emirates; 5 College of Medicine and Health Sciences, Khalifa University of Science and Technology, Abu Dhabi, United Arab Emirates

**Keywords:** Point-of-care ultrasonography, Training, Healthcare, Skills acquisition, Future of work, POCUS

## Abstract

**Background:**

The use of Point-of-Care Ultrasound (POCUS) has become prevalent across a variety of clinical settings. Many healthcare professionals have started getting hands-on training. To evaluate the effectiveness of such training programs, this study aimed to assess a 4 day POCUS training course on healthcare providers’ skills and knowledge acquisition. A secondary objective of this study is to gain valuable insights into the degree of perception, attitude, interest levels and perceived barriers of medical providers performing POCUS.

**Methods:**

This is a prospective cohort study performed on healthcare providers in an integrated healthcare facility in Abu Dhabi undergoing the POCUS training course in February 2022. Course participants took a pre-course survey to evaluate their baseline knowledge, skills, confidence, perception, and interest in POCUS. The same survey was repeated immediately post-course**.** In total, seven healthcare professionals responded to the survey with a response rate of 53.8%. All data and information gathered were used to understand the effectiveness of POCUS training and gain insights into the degree of perception, interest and preparedness of POCUS among healthcare professionals in practice.

**Results:**

Our results demonstrated that the brief POCUS course was effective in improving POCUS skills, knowledge and confidence amongst in-practice healthcare providers from varying medical specialties. The median skill score increased from 25% pre-course to 50% post-course. There is a notable increase in all skills scores after the POCUS training course with the greatest change in scores seen for adjusting ‘gain and depth of image (54.84%), assessing VeXUS score (52.38%) and evaluating lung congestion (50%). The study also provided valuable insights into the perception, attitude, interest and potential barriers of POCUS implementation. Although significant barriers to POCUS are present including the lack of POCUS curriculum, what is challenging is lack of expertise and skills to perform POCUS. Therefore, medical providers must acquire prespecified skills to fully utilize POCUS effectively.

**Conclusion:**

The study confirmed the effectiveness of short POCUS training in improving the skills, knowledge and confidence of medical providers in practice. Healthcare professionals can master POCUS skills and techniques and gain confidence through brief training courses.

**Supplementary Information:**

The online version contains supplementary material available at 10.1186/s13089-023-00350-5.

## Background

With the advancements in hand held ultrasound technology, point-of-care ultrasonography (POCUS) is increasingly used in healthcare by multiple specialities. The term point of care ultrasound indicates a portable ultrasound that can be transported wherever the patient is. POCUS allows clinicians to perform ultrasound imaging directly at the bedside in order to make timely diagnoses [1]. Initially, POCUS was introduced as the focused assessment during trauma and performing bed side procedures [2]. However, with time, it has been increasingly used in other applications due to its portability and accessibility [3]. Today, POCUS could be defined as the stethoscope of the twenty-first century.

With the need to make a timely diagnosis, POCUS has become prevalent across various clinical settings. It is currently being implemented across a wide range of medical specialities by clinicians at the bedside. Clinical use of POCUS includes recognizing pleural and pericardial effusions, evaluation of fluid status and hemodynamic states [4].

There is no doubt that POCUS has the potential to transform healthcare delivery through its expedient assessment [1]. Earlier studies showed that using POCUS in clinical medicine led to improved diagnostic capability, reduced procedural complications and enhanced patient satisfaction [5]. Furthermore, it enables healthcare professionals to evaluate patients in a non-invasive and safe way by not exposing them to harmful ionising radiation. In addition, it can be applied in resource-limited settings, where restricted access to traditional radiological technologies and specialists professionals can be a barrier to care. For example, during transportation to an emergency room, a patient could be scanned using a portable ultrasound device in an ambulance [6].

Despite its potential advantages, POCUS remains underutilized due to several barriers. The most reported barriers to adopting POCUS are operators' lack of adequate skills and experience [7]. The Agency for Healthcare Research and Quality (AHRQ) stated that adequate training is a core safety measure for the adoption of POCUS. For this reason, POCUS has been endorsed as an important clinical skill and appropriate training is required among medical professionals in various specialities.

In the recent years, the adaptation of POCUS extended to residency education. Nevertheless, there is a significant training gap among healthcare providers who started practicing before the integration of POCUS. As a result, these professionals have been seeking training opportunities through national continuing medical education courses that offer practical, hands-on instruction. Although these courses are often brief and short in duration, around 2–4 days, they have improved POCUS skills and proficiency immediately [8, 9].

As the use of POCUS is expanding into medical specialities, it is important to evaluate the skill acquisition. The primary objective of this study is to evaluate the effect of a 4 day POCUS training course on healthcare providers’ skills and knowledge acquisition. A secondary objective of this study is to gain valuable insights into the degree of perception, attitude, interest levels and perceived barriers of medical providers performing POCUS after the training course.

## Methods

A. Study design and ethics approval

This is a prospective cohort study performed on healthcare providers in Sheikh Shakhbout Medical City (SSMC) undergoing the POCUS training workshop. The workshop was held in February 2022 for four days. Before starting the POCUS workshop, all participants took a pre-course survey to evaluate their baseline knowledge, skills, confidence, attitude, perception and interest in POCUS. Afterwards, the training took place in four phases. The first phase dealt with the acquisition of theoretical knowledge. The second and third phases included acquiring basic practical knowledge through workshops, simulators and mannequins. The fourth phase included learning at the bedside with patients in real-world clinical contexts. The first three days of education included 30 min of lectures and video review followed by 60 min of hands-on practice on models to reinforce the techniques. Topics covered included the ultrasound physics, Doppler and knobology (how to use ultrasound device and image optimisation), basic cardiac views, ultrasound of the lungs and systemic veins (Venous Excess Ultrasound, VeXUS), estimation of cardiac output and assessment of fluid responsiveness. The last day included an integrated hands-on training on hemodynamic monitoring followed by a post-course survey to evaluate the knowledge and skills acquisitions as well as the quality of training sessions. The study utilized Kirkpatrick's model, an internationally recognized tool for evaluating training programs, to evaluate the knowledge, skills, and frequency of POCUS usage pre-course and immediately post-course, to assess the outcomes (Level-1 reaction, level-2 learning, level-3 behaviour and level-4 results) [10, 11]. Ethical approval was obtained from the institutional review board of SSMC and the requirement for informed consent was waived due to the deidentified nature of the survey.

B. Study population and settings

Healthcare providers meeting inclusion criteria, with minimal or no prior ultrasound experience from general medicine and critical care, were invited to participate in the 4 day POCUS training course. The training course was initiated on February 15, 2022, which served as pre-course assessment, till February 18, 2022, which served as the post-course assessment. Healthcare professionals were also invited to complete a pre-training and post-training surveys to assess the quality of training sessions. Participation in the survey was on a voluntarily basis and all responses were kept confidential. Participants who completed both, the pre- and post-training surveys, were included in the study and their results were analysed. Those who failed to return both surveys were excluded from the study.

C. Survey development and data collection

Two web-based structured surveys, the pre- and post-course, were developed using the Google Forms software and were sent to the training participants via the hospital’s internal email system (Additional file 1). Medical providers working in SSMC were asked to complete both surveys. The pre-course survey had 3 main components which contained a total of 36 questions including 2 clinical-based questions on image interpretation. It collected information regarding sociodemographic characteristics, including clinical speciality, role, number of years in practice and past experience with POCUS. In addition, the surveys asked questions about (1) the basic understanding of POCUS, (2) former POCUS training, level of exposure and current experience with POCUS, (3) interest and willingness to use POCUS device and (4) perceived potential barriers to the implementation of POCUS. On the other hand, the post-course survey collected data on the (1) usefulness of POCUS, (2) ways to capture the opportunities presented by POCUS and (3) an evaluation of the training curriculum. Further, to assess the knowledge and skills acquisition of POCUS, healthcare providers were asked to self-rate their competency in 16 POCUS applications on a 5-point Likert scale, where 5 = “very confident”, 4 = “confident”, 3 = “neither confident nor not confident”, 2 = “not confident”, and 1 = “not at all confident”, pre-course, on day 1, and immediately post-course, on day 4.

D. Data analysis and assessment tool

The data were analysed using R studio software. Descriptive analysis, such as mean, standard deviation and frequencies were used to describe the study cohort. Visual boxplots were used to investigate any difference in score for skills and knowledge acquisitions from pre-course to post-course. All data and information gathered were used to understand the effectiveness of POCUS training and gain insights into the degree of perception, interest and preparedness of POCUS among practising medical providers.

## Results

A. Survey participants

**Fig. 1:**
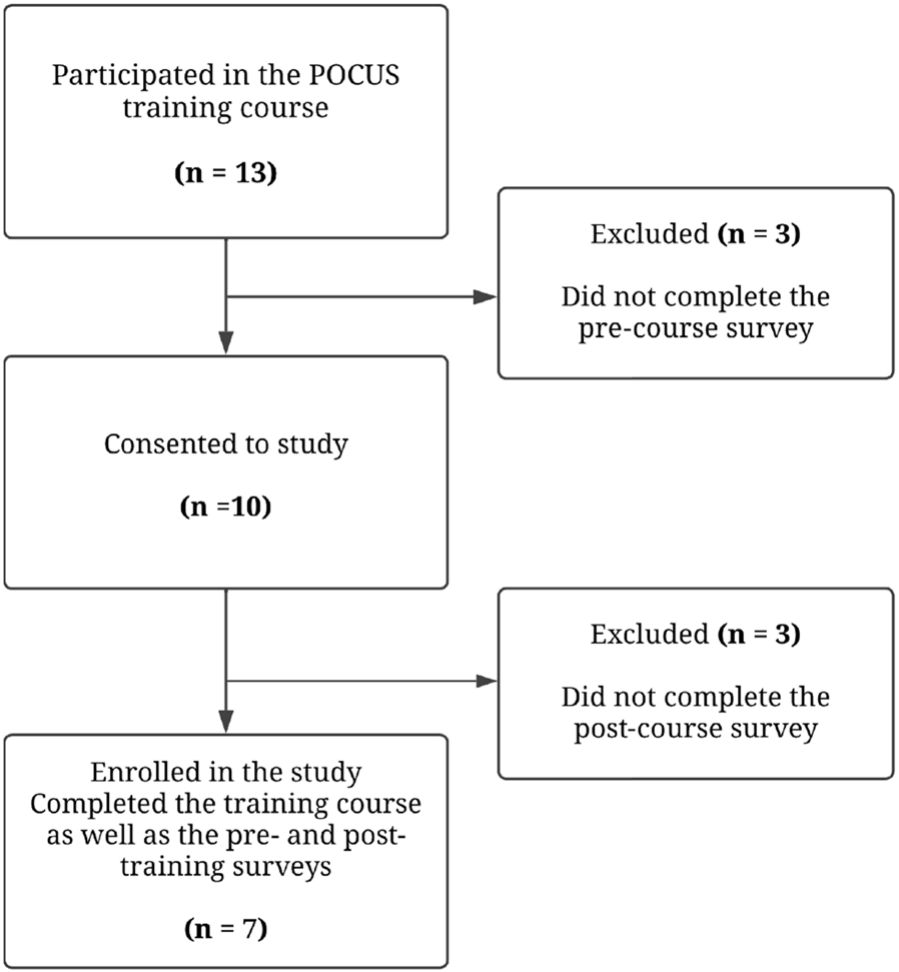
Survey participation flowchart

Overall, 13 healthcare professionals from SSMC participated in the POCUS training course of whom 7 (response rate = 53.8%) completed both surveys and were included in the study as summarized by the flowchart in Fig. 1 Sociodemographics of participants are presented in Table 1.

**Table 1:** Sociodemographic characteristics of the participants included in the study (*N* = 7)

Characteristic	*n* = 7	(%)
Specialty		
General medicine	5	71
Critical care	2	29
Role		
Consultant	2	29
Specialist	1	14
Nurse	1	14
Trainee	3	43
Years in practice		
0–3	1	14
4–10	3	43
> 10	3	43
POCUS experience in patient care		
Yes	2	29
No	5	71
Prior POCUS knowledge/training/education		
Yes	4	57
No	3	43

Respondents median years of experience were 8 years. The majority of respondents came from “General Medicine” (71%) and few came from “Critical Care” (29%). Almost half of the participants were senior doctors with more than 10 years of medical experience.

B. Exposure

The proportion of respondents who have prior knowledge, education or training in POCUS is 57% (*N* = 4). The most quoted form of POCUS education is the residency program. The rest of the respondents had either online courses or exposure through medical conferences. Only 29% of the respondents (*N* = 2) have prior experience with integrating POCUS modalities in their clinical work while the majority have never performed any POCUS.

C. Perception

Overall, all respondents viewed POCUS positively. Respondents perceived that POCUS reduces time to reach diagnosis and allows for procedures to be carried more safely. The majority of respondents agreed that POCUS will improve diagnostic accuracy (*M* = 0.857 STD = 0.378) while only few felt that POCUS will allow for career advancement (*M* = 0.571, STD = 0.535). More than three-quarters of the participants stated that POCUS will have an impact on physical examination and the overall patient care. 1 respondent believed that POCUS is most likely to replace stethoscope. Another respondent claimed that integrating POCUS into the clinical practice would lead to early diagnosis and achieve cost effectiveness. 1 participant believe that the use of POCUS will be most beneficial in emergency and resuscitation situation where accurate and timely diagnosis is needed.

D. Barriers

Significant barriers are present in the adoption of POCUS. According to respondents, the five most identified barriers were (1) No POCUS curriculum (*M* = 0.714, STD = 0.488), (2) No experts for hands-on training and/or maintenance education (*M* = 0.714, STD = 0.488), (3) Lack of confidence in ability to obtain/interpret images (*M* = 0.714, STD = 0.488), (4) No method to provide feedback on image interpretation (*M* = 0.429 STD = 0.535) and (5) No POCUS leader/director (*M* = 0.429 STD = 0.535). Table 2 summarizes all perceived barriers to POCUS teaching and adoption. While all healthcare providers with different years of practice listed the lack of POCUS curriculum as a major barrier to the POCUS implementation, senior-level medical providers were more concerned with the potential interdisciplinary conflicts that POCUS might bring over to other specialities.

E. Course evaluation

**Table 2:** Barriers to POCUS train and use

Barrier	Respondents
No machine	2
No POCUS leader/director	3
No POCUS curriculum	5
No quality assurance/improvement program	2
No accreditation pathway	1
Inadequate institutional support	2
No time	1
Not enough personal interest	1
Lack of confidence in ability to obtain/interpret images	5
No method to provide feedback on image interpretation	3
No experts for hands-on training and/or maintenance education	5
Potential interdisciplinary conflicts over POCUS with other specialties	2

For Kirkpatrick level 1 assessment (Reaction), six healthcare providers who completed the survey were satisfied or very satisfied with the POCUS training, content and instructors. On the other hand, one participant believed the course was irrelevant to their field of practice or their learning objective. Five participants plan to incorporate the knowledge and skills they acquired during the training into their clinical practice, of which, two believe that they need more hands-on training and real-time clinical scenario to maximize the benefits of the training.

F. Skill and knowledge acquisition

**Fig. 2:**
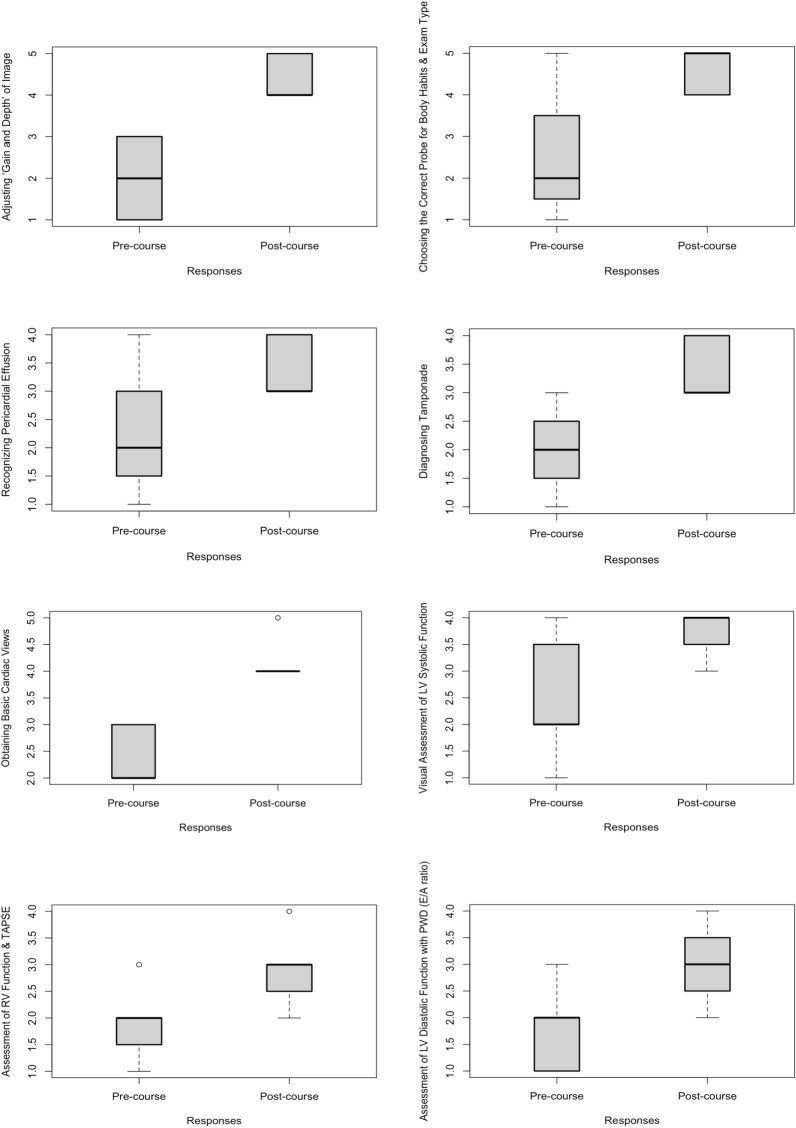

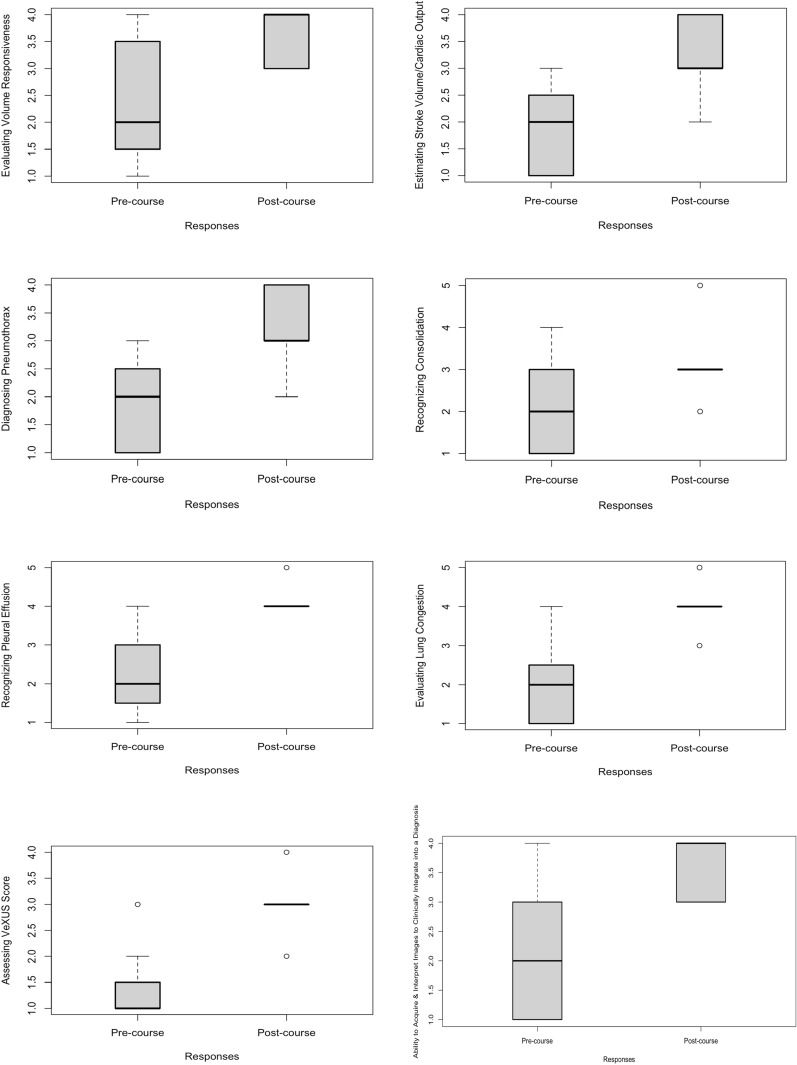
Pre- and Post-Training Self-Assessment of POCUS Knowledge and Skills

The rate of learning for POCUS is assessed using pre- and post-training evaluation on a Likert scale. The results of the survey for pre- and post-training self-ratings are summarized in Tables 3, 4 respectively. Additionally, Appendix 1: Fig. 2 illustrates the boxplots comparing participants' self-assessed knowledge and skills in POCUS, pre- and post-training. The median competency score exhibited a significant rise, from 25% before the training to 50% following its completion. There is a notable increase in all skills scores after the POCUS training course with the greatest change in scores seen for adjusting ‘gain and depth of image (54.84%), assessing VeXUS score (52.38%) and evaluating lung congestion (50%).

**Table 3:** Pre- POCUS training skill competency rating on a 5-point Likert Scale

Participant No	1	2	3	4	5	6	7
Adjusting ‘gain and depth’ of image	3	1	1	1	3	3	2
Choosing the correct probe for body habits & exam type	4	1	1	2	5	3	2
Recognizing pericardial effusion	3	1	1	2	4	3	2
Diagnosing tamponade	2	1	2	1	3	3	2
Obtaining basic cardiac views	3	2	2	2	3	3	2
Visual assessment of LV systolic function	4	2	2	1	4	3	2
Assessment of RV function and TAPSE	2	2	1	1	2	3	2
Assessment of LV diastolic function with PWD (E/A ratio)	2	1	1	1	2	3	2
Evaluating volume responsiveness	4	1	2	1	4	3	2
Estimating stroke volume/cardiac output	3	1	1	1	2	3	2
Diagnosing pneumothorax	3	1	1	1	2	3	2
Recognizing consolidation	4	1	1	1	3	3	2
Recognizing pleural effusion	3	1	2	1	4	3	2
Evaluating lung congestion	4	1	1	1	2	3	2
Assessing VeXUS score	1	1	1	1	1	3	2
Ability to acquire and interpret images to clinically integrate into a diagnosis	4	1	1	1	3	3	2

5 = “very confident”, 4 = “confident”, 3 = “neither confident nor not confident”, 2 = “not confident”, and 1 = “not at all confident”

**Table 4:** Post POCUS training skill competency rating on a 5-point Likert Scale

Participant No	1	2	3	4	5	6	7
Adjusting ‘gain and depth’ of image	4	4	5	4	4	5	5
Choosing the correct probe for body habits & exam type	4	4	5	4	5	5	5
Recognizing pericardial effusion	3	3	3	3	4	4	4
Diagnosing tamponade	3	3	4	3	3	4	4
Obtaining basic cardiac views	4	4	4	5	4	4	4
Visual assessment of LV systolic function	4	3	4	4	4	3	4
Assessment of RV function and TAPSE	3	2	3	3	3	2	4
Assessment of LV diastolic function with PWD (E/A ratio)	4	2	4	3	3	2	3
Evaluating volume responsiveness	3	3	4	4	4	3	4
Estimating stroke volume/cardiac output	3	2	4	3	4	3	4
Diagnosing pneumothorax	3	3	3	4	2	4	4
Recognizing consolidation	3	3	3	3	2	5	3
Recognizing pleural effusion	4	4	4	4	4	5	4
Evaluating lung congestion	4	3	4	4	4	5	4
Assessing VeXUS score	3	3	3	2	3	4	3
Ability to acquire and interpret images to clinically integrate into a diagnosis	4	3	4	3	3	4	4

5 = “very confident”, 4 = “confident”, 3 = “neither confident nor not confident”, 2 = “not confident”, and 1 = “not at all confident”

When asked to state the areas of POCUS they would like to improve their diagnostic confidence, the majority of participants listed focused cardiac (85.7%), IVC volume assessment (85.7%) and lung ultrasound (71.4%) as their main areas of improvement. Only few stated that they would like to further develop in E-FAST, procedural and soft tissue POCUS areas.

## Discussion

Our study utilized the Kirkpatrick’s model for evaluating the brief POCUS training course in a large tertiary care academic healthcare facility in Abu Dhabi, UAE. The evaluation model focused on the first two levels, Level 1—Reaction, to assess how participants responded to and felt about the training and Level 2-Learning, to measure the degree to which participants have improved their knowledge, skills and confidence from pre-course to post-course. The results demonstrated that, overall, participants found the training experience valuable and relevant (Level 1) and were able to improve their POCUS knowledge, skills and confidence (Level 2). A significant increase in skills competency was seen for all participants from pre-course to immediately post-course indicating that short POCUS training program is indeed adequate in improving the knowledge and skills among practicing medical providers, similar to other studies [12]. This emphasizes the need to implement POCUS training and education into all medical programs. Our results also showed that the course was effective in improving POCUS knowledge and confidence amongst in-practice healthcare providers from varying medical specialties, consistent with those of previous published POCUS studies [13].

Moreover, the results of the survey provided valuable insights into the level of interest, perception, attitude, exposure, and potential barriers to POCUS. First, POCUS is not novel to healthcare professionals who underwent the survey. More than half had prior knowledge, training and education in POCUS, however, only a few practiced POCUS before the course. Mainly, participants received POCUS formal training in their residency program whereas few had informal training either via online courses or through attending conferences. Secondly, although most participants were not exposed to POCUS in their clinical practice, most of them viewed POCUS positively.

Improving diagnostic accuracy, allowing procedures to be carried out safely and achieving cost effectiveness are rated by course participants as the most beneficial outcomes of POCUS. Moreover, the level of participants’ interest in undergoing further trainings in POCUS was found very high. This positive perception and interest lay a good foundation for healthcare policymakers and provide an opportunity to start the adoption of POCUS and transform healthcare.

Our findings also provided an identification of perceived barriers to the adoption of POCUS. The most identified barrier by participants is the lack of POCUS curriculum, emphasizing the need to develop a standardized POCUS curriculum for practicing healthcare providers. Similar observations were made in other studies [14]. Furthermore, the lack of confidence in the ability to obtain/interpret images is also a significant deterrent to the use of POCUS among healthcare providers. Misinterpretation may lead to a diagnostic error which thereby raises serious issues and potentially delay proper treatment if diagnosis is missed, delayed or wrong (6). This barrier can be overcome by developing POCUS skills which includes probe handling and improving hand–eye coordination skills. Another remarkable insight gained is that the lack of personal interest was not a significant barrier to the training nor to the POCUS use. This indicates their preparedness for the utilization of POCUS in their everyday work. Additionally, the lack of expert mentors for hand-on training and ongoing education may be an obstacle which jeopardizes the efficiency of POCUS adoption. To this reason, healthcare systems must invest heavily in acquiring external expertise to train and evaluate POCUS competency, especially in low-and middle-income countries with limited resources and skilled experts [15].

To the best of our knowledge, this study is the first study in the Middle East region to evaluate the efficacy of POCUS training program. Besides, our study evaluated the level of interest, perception and attitude of practicing medical providers towards the use of POCUS which is informative for policymaking decision. Moreover, previous studies are limited to reporting the effect of POCUS training on medical students and trainees only as course participants and not practicing healthcare providers. Our study, however, focuses on practicing providers as well as trainees. Widespread integration of POCUS in clinical practice require different training programs for medical providers at a different stage of clinical practice.

## Limitations

This study has several limitations that we intend to address in future studies. First, this study is limited by size (*n* = 7) and reflect a relatively low number of healthcare providers in the United Arab Emirates. The response rate of 53% could be improved by providing various incentives, though for the purpose of the study, no incentives were provided. Secondly, the use of single healthcare facility, SSMC, might have biased the results towards higher POCUS interest, attitude and perception. Thus, the results may not be easily generalizable to the whole population of healthcare professionals. Furthermore, the study explored only conceptual and self-rated knowledge, skills and confidence in performing POCUS. Instead, skills and knowledge tests must be conducted where participants are assessed by scores based on the performance of prespecified tasks. The study is also limited to assessment of skill acquisition immediately after the training course and does not assess the long-term retention of skills. Further studies should focus on assessing the POCUS skill retention after 6, 9 and 12 months.

## Conclusion

POCUS, an emerging diagnostic tool, serves as a valuable addition to enhance bedside physical examination in clinical practice. It offers several advantages, including safety, cost-efficiency, and the potential to enhance diagnostic capabilities in the field of clinical medicine. The use of POCUS has become prevalent across a variety of clinical settings and nowadays it is considered an important clinical skill. Although POCUS curricula have been extended to medical schools and residency programmes, a gap remains among physicians who entered practice before POCUS integration became widespread.

Existing research proposes that physicians might achieve POCUS proficiency with minimal training. However, based on our small sample size, our study provides tentative indications of the effectiveness of short POCUS training in improving the skills, knowledge and confidence of medical providers in practice. Thus, while healthcare professionals appear to exhibit the potential to master POCUS skills and gain confidence through brief training courses, further extensive research is warranted to validate these findings.

The study further offers valuable insights into the perception, attitude, interest and potential barriers of POCUS implementation, such as the lack of POCUS curriculum and limited expertise and skills to perform POCUS. To this reasons, medical providers must acquire prespecified skills to use POCUS effectively and accurately. Based on the findings of the study, healthcare professionals are interested, prepared and ready for POCUS learning and implementation however, the workforce must be trained to be fully equipped with the necessary skills to be able to utilize the opportunities offered and perform POCUS safely at the bedside. This study has helped us to create a comprehensive POCUS training program in a first of its kind initiative in the Middle East region to help integrate its use in clinical practice [16]. In addition, this study may serve as a basic assessment to healthcare policymakers in the future implementation of POCUS. Given the limitations of our study, large-scale research is essential to support the widespread implementation of POCUS training in healthcare.

### Supplementary Information

**Additional file 1:** Post-Course Questionnaire for POCUS Training – This comprehensive questionnaire evaluates participants' satisfaction with the POCUS Haemodynamics Training course, their confidence in various POCUS techniques, and their perspectives on the future integration of POCUS in healthcare, using a mix of Likert scale ratings and open-ended questions.

**Additional file 2: **Pre-Course Questionnaire for POCUS Training – This questionnaire is to assess participants' baseline knowledge and skills in Point-of-Care Ultrasound (POCUS) before undergoing the training course. It includes questions to gauge their initial confidence and proficiency in various POCUS techniques and applications.

## Data Availability

The datasets used and/or analysed during the current study are available from the corresponding author on reasonable request.

## Appendix 1

See Fig. 2

## Abbreviations

POCUS: Point-of-care ultrasound
SSMC: Sheikh Shakhbout Medical City
E-FAST: Extended focused assessment for trauma
AHRQ: Agency for healthcare research and quality
