# Supplementary material for: Evaluation of point-of-care ultrasound training among healthcare providers: a pilot study
Source: Ultrasound J. 2024 Feb 21;16:12. doi: 10.1186/s13089-023-00350-5 (PMC10881927; doi:10.1186/s13089-023-00350-5)
Supplement: Supplementary file 1 — Additional file 1: Post-Course Questionnaire for POCUS Training – This comprehensive questionnaire evaluates participants' satisfaction with the POCUS Haemodynamics Training course, their confidence in various POCUS techniques, and their perspectives on the future integration of POCUS in healthcare, using a mix of Likert scale ratings and open-ended questions. [file 13089_2023_350_MOESM1_ESM.pdf]

EchoNous / SSMC POCUS Haemodynamics Training
Abu Dhabi, UAE

Post-Course Questionnaire

This section deals with the first level of Kirkpatrick Evaluation Model to assist us in the provision of effective training conditions. Please take few minutes to express your opinion and rate the below questions accurately. All responses will be kept confidential. You will not be identified in any related reports or publications. It is important that you complete all questions.

Please circle the number that best corresponds to your response:

	Strongly Dissatisfied	Dissatisfied	Neutral	Satisfied	Strongly Satisfied
**Instructor Assessment:**					
1. I was satisfied with the course didactics	1	2	3	4	5
2. I was satisfied with the hands-on learning modules	1	2	3	4	5
3. In general, I was satisfied with the course instructors	1	2	3	4	5

Please rate your satisfaction with the course instructors

	Strongly Dissatisfied	Dissatisfied	Neutral	Satisfied	Strongly Satisfied
**Instructor Assessment:**					
1. Hatem Soliman	1	2	3	4	5
2. Fernando Maravilla	1	2	3	4	5

Please circle the number that best characterizes your response:

	Strongly Disagree	Disagree	Neutral	Agree	Strongly Agree
**Instructor Assessment:**					
1. The course was effective in achieving my learning objectives	1	2	3	4	5
2. The course content was relevant to my field of practice	1	2	3	4	5
3. I plan to incorporate the content I learned into my practice	1	2	3	4	5

Please select which training sessions you attended (Select all that apply)

a. Day 1: POCUS Heart
b. Day 2: POCUS Lungs
c. Day 3: POCUS Systemic Veins (VeXUS)
d. Day 4: Integrated Approach

What could be improved with the didactics?
____

What could be improved with the hands-on learning modules?
____

Short Answer Questions
This section of the questionnaire contains a series of open-ended questions about your thoughts regarding the impact of POCUS on the future of the healthcare.

1. How do you think POCUS will change the way you work?
   ____
   ____
   ____

2. Do you believe POCUS will have an impact on physical examination??
   a. No
   b. Yes (specify): ___________________

3. What are the best ways of capturing the opportunities presented by POCUS? Please Explain
   ____
   ____
   ____

4. Do you believe POCUS can be used remotely to treat patients who are self-monitoring at home?
   a. No
   b. Yes (specify): _________________________

5. Do you think POCUS can be useful in any of the following: (select all that applies)?
   a. Improves diagnostic accuracy
   b. Decreases complication rates
   c. Allows for a procedure to be carried more safely
   d. Reduces the time taken to reach diagnosis
   e. Allows for career advancement
   f. None of the above

This section deals with the second level of Kirkpatrick Evaluation Model and will be used for quality improvement and/or research purposes to improve point-of-care-ultrasound education

Questions below ask about your **confidence in using POCUS in patient management.** A Likert Scale is used, with **1** representing 'Not at all confident', **2** 'Not confident', **3** 'Neither confident nor not confident', **4** 'Confident', and **5** 'Very confident'.

Please circle the number that best corresponds to your response:

	Not confident at all	Not confident	Neither	Confident	Very confident
1. Adjusting 'gain and depth' of image?	1	2	3	4	5
2. Choosing the correct probe for body habits & exam type?	1	2	3	4	5
3. Recognizing pericardial effusion?	1	2	3	4	5
4. Diagnosing tamponade?	1	2	3	4	5
5. Obtaining basic cardiac views?	1	2	3	4	5
6. Visual assessment of LV systolic function?	1	2	3	4	5
7. Assessment of RV function and TAPSE	1	2	3	4	5
8. Assessment of LV diastolic function with PWD (E/A ratio)	1	2	3	4	5
9. Evaluating volume responsiveness?	1	2	3	4	5
10. Estimating stroke volume / cardiac output	1	2	3	4	5
11. Diagnosing pneumothorax?	1	2	3	4	5
12. Recognizing consolidation?	1	2	3	4	5

13 Recognizing pleural effusion?	1	2	3	4	5
14. Evaluating lung congestion	1	2	3	4	5
15. Assessing VeXUS score	1	2	3	4	5
16. Ability to acquire and interpret images to clinically integrate into a diagnosis?	1	2	3	4	5

10. Clinical case 1:

You are the medical registrar on the nephrology ward and you are called to see a 65 years old patient who was just admitted with acute on chronic renal impairment and she had dropping blood pressure despite normal gas exchange with worsening acidaemia and hyperlactatemia. On POCUS assessment, she had hyperdynamic LV systolic function, LVOT VTI 20 cm with an IVC diameter of 1 cm, collapsible IVC and lung ultrasound showed Predominant A-lines. VeXUS assessment showed monophasic portal vein. Flow using PWD and hepatic venous flow showing (S>D wave). In this lady, the POCUS findings indicate (choose one option)?

a. systemic venous congestion
b. Hypovolaemia
c. low cardiac output
d. b and c
e. none of the above

11. Clinical case 2:

You are the intensive care registrar oncall and you are called to see a gentleman who had road traffic accident 10 days ago and currently intubated and ventilated. He is warm peripherally with lactate level of 5 mmol/L with worsening metabolic acidaemia. POCUS assessment of the lungs showed the following (image below) and heart POCUS showed LVOT VTI 25 cm with small hyperdynamic LV and normal RV function.

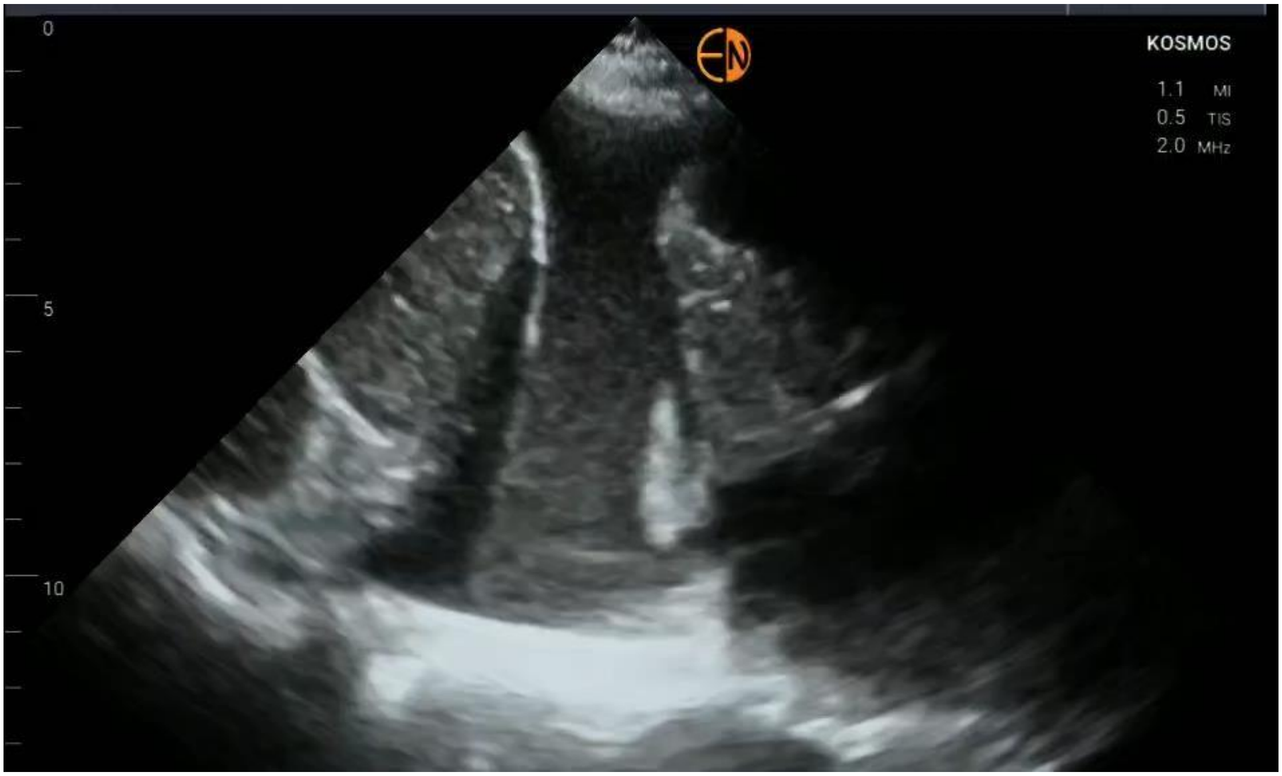

What is the likely diagnosis?

a. Cardiogenic shock
b. Septic shock
c. Obstructive shock
d. Hypovolaemic shock
e. None of the above

12. Clinical case 3: You are the medical registrar oncall and you are called to see a 71 years old lady who underwent right hip replacement surgery 10 days ago. She was cold peripherally with lactate level of 7 mmol/L with worsening metabolic acidaemia. POCUS assessment of the heart showed the following parasternal short axis view (image below) with LVOT VTI 10 cm.

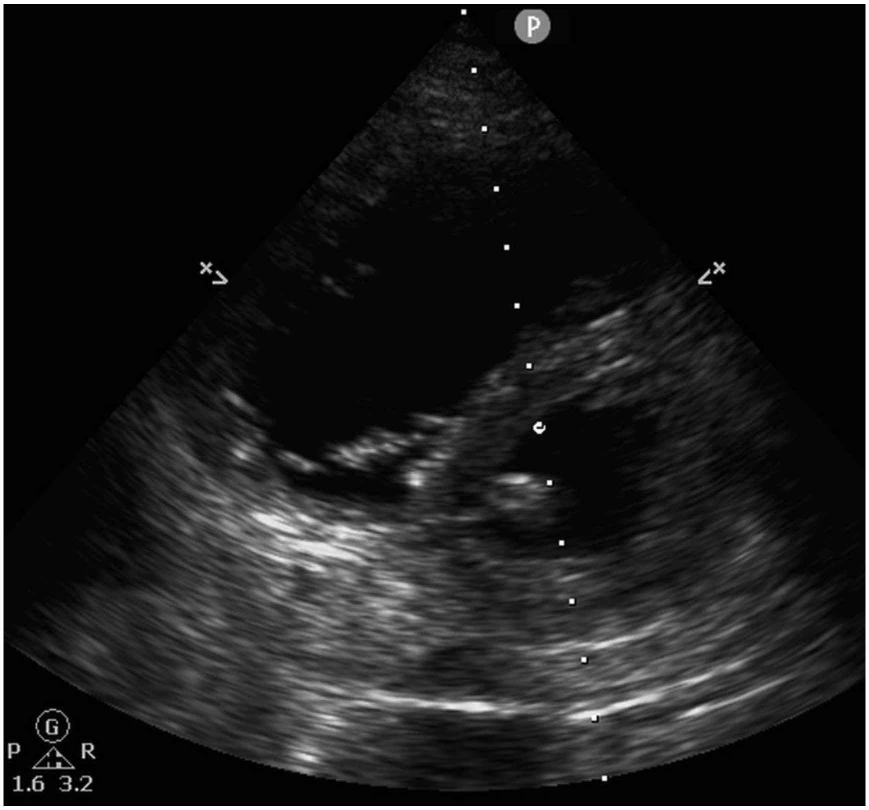

What is the likely diagnosis?

a. Cardiogenic shock
b. Septic shock
c. Obstructive shock due to pulmonary embolism
d. Hypovolaemic shock
e. None of the above
