# Supplementary material for: Evaluation of point-of-care ultrasound training among healthcare providers: a pilot study
Source: Ultrasound J. 2024 Feb 21;16:12. doi: 10.1186/s13089-023-00350-5 (PMC10881927; doi:10.1186/s13089-023-00350-5)
Supplement: Supplementary file 2 — Additional file 2: Pre-Course Questionnaire for POCUS Training – This questionnaire is to assess participants' baseline knowledge and skills in Point-of-Care Ultrasound (POCUS) before undergoing the training course. It includes questions to gauge their initial confidence and proficiency in various POCUS techniques and applications. [file 13089_2023_350_MOESM2_ESM.pdf]

EchoNous / SSMC POCUS Haemodynamics Training
Abu Dhabi, UAE

Course Questionnaire

This questionnaire will be used for quality improvement and/or research purposes to improve point-of-care-ultrasound education. The questionnaire will be administered today, with an electronic follow-up questionnaire and at 3 months, 6 months, and 12 months' time periods. It will take approximately 3-5 minutes to complete this questionnaire and less time for follow-up questionnaires. All responses will be kept confidential. You will not be identified in any related reports or publications. It is important that you complete all questions. Thank you for your participation.

Pre-Course Questionnaire

Questions below ask about your **confidence in using POCUS in patient management.** A Likert Scale is used, with **1** representing 'Not at all confident', **2** 'Not confident', **3** 'Neither confident nor not confident', **4** 'Confident', and **5** 'Very confident'.

Please circle the number that best corresponds to your response:

	Not confident at all	Not confident	Neither	Confident	Very confident
1. Adjusting 'gain and depth' of image?	1	2	3	4	5
2. Choosing the correct probe for body habits & exam type?	1	2	3	4	5
3. Recognizing pericardial effusion?	1	2	3	4	5
4. Diagnosing tamponade?	1	2	3	4	5
5. Obtaining basic cardiac views?	1	2	3	4	5
6. Visual assessment of LV systolic function?	1	2	3	4	5
7. Assessment of RV function and TAPSE	1	2	3	4	5
8. Assessment of LV diastolic function with PWD (E/A ratio)	1	2	3	4	5
9. Evaluating volume responsiveness?	1	2	3	4	5
10. Estimating stroke volume / cardiac output	1	2	3	4	5
11. Diagnosing pneumothorax?	1	2	3	4	5
12. Recognizing consolidation?	1	2	3	4	5

13 Recognizing pleural effusion?	1	2	3	4	5
14.Evaluating lung congestion	1	2	3	4	5
15.Assessing VeXUS score	1	2	3	4	5
16.Ability to acquire and interpret images to clinically integrate into a diagnosis?	1	2	3	4	5

Please circle the answer that best corresponds to your response:

1. Approximately how many patients have you scanned in the last month:
    a. 0
    b. 1-2
    c. 3-4
    d. >4

2. Approximately how many patients have you scanned in the last 3 months:
    a. 0
    b. 1-3
    c. 4-7
    d. >7

3. Approximately how many patients have you scanned in the last 6 months:
    a. 0
    b. 1-4
    c. 5-8
    d. 9-12
    e. >12

4. Approximately how many patients have you scanned in the last 12 months:
    a. 0
    b. 1-5
    c. 6-10
    d. 11-15
    e. >15

5. What scans do you typically perform?
    a. Focused lung ultrasound
    b. Focused cardiac ultrasound
    c. Renal ultrasound

d. FAST
e. Soft Tissue
f. Ultrasound for procedural guidance
g. Other (specify): ________________________

6. What are the barriers that inhibit you or your colleagues from integrating POCUS in your clinical assessment? (Select all that apply)

    a. No machine
    b. No POCUS leader / director
    c. No POCUS curriculum
    d. No quality assurance/improvement program
    e. No accreditation pathway
    f. Inadequate institutional support
    g. No time
    h. Not enough personal interest
    i. Lack of confidence in ability to obtain/interpret images
    j. No method to provide feedback on image interpretation (e.g., KOSMOS UP)
    k. No experts for hands-on training and/or maintenance education
    l. Potential interdisciplinary conflicts over POCUS with other specialties
    m. Other: ___________________________________

7. What could be done to maximize your use of POCUS? ________________________

8. What could be done to maximize the use POCUS by your colleagues? ____ ____

Please provide some information about yourself.
The information you provide is strictly confidential. You will in no way be identified in study-related reports or publications.

1. Specialty or location of work?
    a. General Medicine
    b. Nephrology
    c. Critical Care
    d. Emergency
    e. Other (specify): _______________

2. Role:
    a. Consultant
    b. Specialist
    c. Trainee
    d. Student
    e. Other (specify): ________________

3. Years in practice: __________________

4. On average, how many patients do you look after in a week?
    a. <10
    b. 11-20
    c. 21-30
    d. ≥ 31
    e. 0ther:

5. Do you have prior experience with Point of care ultrasound (POCUS)?

    (Yes/No)

6. What prior training do you have with Point of care ultrasound (POCUS)?
(Select all that apply)

    a. Residency
    b. Fellowship
    c. Conference: ___________________
    d. Simulation lab: _________________
    e. Face to face course:_____________
    f. Online course: _________________
    g. Other: ________________________

7. How many years of experience do you have in performing POCUS? ________________

8. In which areas of POCUS would you like to improve on diagnostic confidence in your practice?
(Select all that apply)

    a. E-FAST
    b. Focused cardiac
    c. IVC volume assessment
    d. Lung
    e. Soft Tissue
    f. Procedural
    g. Other (specify):
    ____

9. What could be done to help you personally use POCUS?
    ____

10. What suggestions or feedback do you have to help you, or your colleagues to use POCUS?
    ____
    ____
    ____

11. Clinical case 1:

You are the medical registrar on the nephrology ward and you are called to see a 65 years old patient who was just admitted with acute on chronic renal impairment and she had worsening shortness of breath despite blood pressure and haemodynamics. On POCUS assessment, she had preserved biventricular systolic function with an IVC diameter of 2.5 cm and lung ultrasound showed bilateral coalescent symmetrical B lines with thin pleural lines. VeXUS assessment showed portal vein pulsatility (>50%) and systolic reversal of hepatic vein flow with isolated diastolic flow in the renal vein by pulsed wave Doppler. In this lady.

The POCUS findings indicate (choose one option)?

a. systemic venous congestion
b. pulmonary congestion
c. low cardiac output
d. and and b
e. all of the above

12. Clinical case 2:

You are the intensive care registrar oncall and you are called to the accident and emergency department to see a man who had a road traffic accident with pelvic fracture. He is drowsy, cold and clammy peripherally with lactate level of 6 mmol/L with metabolic acidaemia. POCUS assessment showed small hyperdynamic LV systolic function with normal RV function. IVC was 1 cm and collapsible with respiration > 50% with monophasic portal venous flow by PWD and hepatic venous flow showing S/D ratio > 1. Lung ultrasound showed predominant A lines bilaterally with preserved pleural sliding.

What is the likely diagnosis?

a. Cardiogenic shock
b. Septic shock
c. Obstructive shock
d. Hypovolaemic shock
e. None of the above
